# Simultaneous Mass Spectrometry-Based Apolipoprotein Profiling and Apolipoprotein E Phenotyping in Patients with ASCVD and Mild Cognitive Impairment

**DOI:** 10.3390/nu14122474

**Published:** 2022-06-15

**Authors:** Ilijana Begcevic Brkovic, Benedikt Zöhrer, Markus Scholz, Madlen Reinicke, Julia Dittrich, Surab Kamalsada, Ronny Baber, Frank Beutner, Andrej Teren, Christoph Engel, Kerstin Wirkner, Holger Thiele, Markus Löffler, Steffi G. Riedel-Heller, Uta Ceglarek

**Affiliations:** 1 Institute of Laboratory Medicine, Clinical Chemistry and Molecular Diagnostics, Leipzig University, 04103 Leipzig, Germany; ilijana.begcevic@medizin.uni-leipzig.de (I.B.B.); madlen.reinicke@medizin.uni-leipzig.de (M.R.); julia.dittrich@medizin.uni-leipzig.de (J.D.); surab.kamalsada@medizin.uni-leipzig.de (S.K.); ronny.baber@medizin.uni-leipzig.de (R.B.); 2 Respiratory Medicine Unit, Department of Medicine Solna and Center for Molecular Medicine, Karolinska Institute, 17174 Solna, Sweden; benedikt.zohrer@ki.se; 3 Institute for Medical Informatics, Statistics and Epidemiology, Leipzig University, 04107 Leipzig, Germany; markus.scholz@imise.uni-leipzig.de (M.S.); christoph.engel@imise.uni-leipzig.de (C.E.); kerstin.wirkner@medizin.uni-leipzig.de (K.W.); markus.loeffler@imise.uni-leipzig.de (M.L.); 4 LIFE—Leipzig Research Center for Civilization Diseases, Leipzig University, 04103 Leipzig, Germany; beutner.le@googlemail.com (F.B.); andrej.teren@medizin.uni-leipzig.de (A.T.); 5 Department of Internal Medicine/Cardiology, Heart Center Leipzig, University of Leipzig, 04289 Leipzig, Germany; holger.thiele@medizin.uni-leipzig.de; 6 Department of Cardiology and Intensive Care Medicine, University Hospital OWL, Campus Klinikum Bielefeld, 33604 Bielefeld, Germany; 7 Institute of Social Medicine, Occupational Health, and Public Health, University of Leipzig, 04103 Leipzig, Germany; steffi.riedel-heller@medizin.uni-leipzig.de

**Keywords:** apolipoproteins, apolipoprotein E phenotype, liquid chromatography, mass spectrometry, proteomics

## Abstract

Apolipoprotein E (apoE) occurs on the majority of plasma lipoproteins and plays a major role in the lipid metabolism in the periphery and in the central nervous system. ApoE is a polymorphic protein with three common isoforms, apoE2, apoE3 and apoE4, derived from respective alleles ε2, ε3 and ε4. The aim of this study was to develop a sample pretreatment protocol combined with rapid mass spectrometry (MS)-based assay for simultaneous apolipoprotein profiling and apoE phenotype identification. This assay was validated in 481 samples from patients with stable atherosclerotic cardiovascular disease (ASCVD) and applied to study association with mild cognitive impairment (MCI) in the LIFE Adult study, including overall 690 study subjects. Simultaneous quantification of 8–12 major apolipoproteins including apoA-I, apoB-100 and apoE could be performed within 6.5 min. Phenotyping determined with the developed MS assay had good agreement with the genotyping by real-time fluorescence PCR (97.5%). ApoE2 isoform was associated with the highest total apoE concentration compared to apoE3 and apoE4 (*p* < 0.001). In the subgroup of diabetic atherosclerotic cardiovascular disease (ASCVD) patients, apoE2 isoform was related to higher apoC-I levels (apoE2 vs. apoE3, *p* < 0.05), while in the subgroup of ASCVD patients under statin therapy apoE2 was related to lower apoB-100 levels (apoE2 vs. apoE3/apoE4, *p* < 0.05). A significant difference in apoE concentration observed between mild cognitive impairment (MCI) subjects and controls was confirmed for each apoE phenotype. In conclusion, this study provides evidence for the successful implementation of an MS-based apoE phenotyping assay, which can be used to assess phenotype effects on plasma lipid and apolipoprotein levels.

## 1. Introduction

Lipoproteins are the primary transporters of cholesterol and fatty acids in circulation. On the surface of lipoproteins are located apolipoproteins (apo), responsible for lipid transportation and interaction with specific receptors, facilitating uptake and deposition of lipids into the tissue. Human apolipoprotein E (apoE) is a 299 amino acid glycoprotein predominantly synthesized by hepatocytes in the liver and astrocytes in the central nervous system [1,2]. ApoE mediates lipid, cholesterol and lipoprotein transport and metabolism by binding to a variety of lipoprotein receptors [3,4,5]. It occurs on the majority of plasma lipoproteins such as chylomicron remnants, very-low-density lipoproteins (VLDL), intermediate-density lipoproteins (IDL), low-density lipoproteins (LDL) and high-density lipoproteins (HDL).

ApoE has three isoforms apoE2, apoE3 and apoE4, encoded by respective alleles of the *APOE* gene, ε2, ε3 and ε4, with a frequency of 7, 79 and 14% in the general population, respectively [6]. Isoforms differ in merely two amino acid positions: Cys112 and Cys158 in apoE2; Cys112 and Arg158 in apoE3; Arg112 and Arg158 in apoE4 [7]. The three homozygous and heterozygous genotypes have the following distribution in the population: ε2/ε2 0.7%, ε2/ε3 11.0%, ε2/ε4 1.9%, ε3/ε3 62.3%, ε3/ε4 22.2% and ε4/ε4 1.9% [8]. The clinical interest in *APOE* polymorphism is mainly based on the differential risk for developing cardiovascular, neurological, and infectious diseases. Carriers of two ε4 alleles have a 9–15 fold risk of developing late-onset Alzheimer’s disease (AD) [9]. On the contrary, the ε2 allele seems to have a protective effect on AD [10]. Apart from AD, the ε4 allele is associated with an increased risk for cardiovascular disease, while homozygous ε2 carriers have a higher risk for developing type III hyperlipoproteinemia [11]. Polymorphic amino acid changes result in functional differences between apoE isoforms, affecting their binding to receptors and lipids. For instance, apoE2 has a lower affinity to LDL receptors compared to apoE3 and apoE4 [12]. It has been demonstrated that total apoE concentration in human plasma is strongly influenced by the three *APOE* alleles. The highest apoE levels were observed in *APOE* ε2/ε2 carriers, while ε4/ε4 carriers showed the lowest apoE plasma concentrations [13,14,15].

In past years, different targeted liquid chromatography-tandem mass spectrometry (LC-MS/MS) methods were published with the aim to identify and/or quantify apoE phenotypes, monitoring specific peptides for each isoform [16,17,18,19]. Yet, there are very limited LC-MS/MS assays designed to simultaneously define apoE phenotypes and quantitatively measure plasma apolipoproteins [17,20]. In fact, such a multiplexed high-throughput method is an unmet clinical need for improved cardiovascular risk assessment. Previously, our group developed a rapid multiplexed LC-MS/MS assay for quantitative analysis of an apolipoprotein panel in human plasma and found total apoE plasma concentrations to be independently associated with stable atherosclerotic cardiovascular diseases in the Leipzig LIFE-Heart Study [21]. This urged us to further investigate the association of apoE isoforms with atherosclerotic cardiovascular disease by developing a method combining apolipoprotein profiling and apoE phenotyping. Although the impact of *APOE* genotypes on plasma apoE levels, as well as on lipids and lipoprotein concentrations, has been investigated before, less is known about the associations of *APOE* genotypes with other plasma apolipoprotein species. Therefore, the aims of this study were: (a) the implementation of qualitative apoE phenotyping into our quantitative apolipoprotein LC-MS/MS assay; (b) the investigation of relationships between total apoE plasma concentrations and apoE phenotypes in patients with stable coronary artery disease and patients with mild cognitive impairment; (c) the evaluation of effects of apoE phenotypes on different plasma apolipoprotein and lipid species.

## 2. Materials and Methods

### 2.1. Study Design and Participants

The LIFE-Heart and LIFE-Adult are observational studies of individuals recruited at the Heart Center Leipzig at the University of Leipzig and the LIFE Leipzig Research Centre for Civilization Diseases, Germany, respectively [22,23]. Both studies were approved by the Ethics Committee of the Faculty of Medicine, Leipzig University, Germany (Reg. No 276-2005 and 263-2009-14122009, respectively) and all participants signed the written informed consent. The samples were stored either at −80 °C (LIFE-Heart Study) or in the vapor phase of liquid nitrogen (LIFE-Adult Study) in the Leipzig Medical Biobank. For the present study, we used a subset of patients from the LIFE-Heart study with known *APOE* genotype and stable stenosis ≥ 50% in one or more major coronary arteries (diagnosed as coronary artery disease, CAD) and patients with angiographically normal coronary arteries (control group) for method validation. For the association of apoE phenotypes with cognitive impairment, individuals from the population-based LIFE-Adult study above the age of 60 years, who underwent intensive cognitive testing, were recruited, with existing SISCO (SIDAM (Structured Interview for the diagnosis of Dementia of the Alzheimer type, Multi-infarct dementia and dementias of other etiology according to ICD-10 and DSM-III-R)-Score) and the MMSE (Mini-Mental State Examination) scores. Participants with a SISCO score ≤ 51 were defined as mildly cognitively impaired (MCI) [24], whereas the participants with a SISCO > 51 as controls with normal cognitive function. Individual *APOE* genotypes (details on analysis are described below) were enriched among participants to obtain a more equal *APOE* genotype distribution. Finally, 481 subjects of the LIFE-Heart (in the following text CAD cohort) and 209 subjects of the LIFE-Adult Study (in the following text MCI cohort) were included in the statistical analyses. For both study cohorts, individuals with renal impairment (serum creatinine concentration > 132 μmol/L) and incomplete medical history documentation were excluded. Demographic and clinical characteristics of CAD and MCI cohorts are described in Supplementary Table S1.

### 2.2. LC-MS/MS Analyses

LC-MS/MS analysis was based on our in-house protocol for multiplex apolipoprotein quantification [25,26]. A detailed description of the method is given in the Supplementary Materials. In brief, 3 µL of EDTA-plasma, calibration standards or quality controls were 2-fold diluted with stable isotope-labeled (SIL) peptides in ammonium bicarbonate buffer. Protein denaturation was performed with 2,2,2-trifluoroethanol followed by disulfide reduction with tris(2-carboxyethyl)phosphine (TCEP). Cystein peptide residues were alkylated with iodoacetamide (IAM) (LIFE Heart) or N-ethylmaleimide (NEM) (LIFE Aldult). Tryptic digestion was finished after 16 h at 37 °C. A total of 5 µL of the sample was injected into the LC-MS/MS system consisting of an Ultimate 3000 RSLCnano System (Thermo Scientific Dionex, Sunnyvale, CA, USA) coupled with a QTRAP 5500 mass spectrometer (SCIEX, Darmstadt, Germany), operating in positive ESI mode. Samples were analyzed within a total run time of 6.5 min applying on-line solid-phase extraction (SPE) coupled with reversed-phase chromatography. The unlabeled and labeled peptide standards were provided by the Core Unit Peptide Technologies Leipzig (Faculty of Medicine, Leipzig University, Leipzig, Germany). Monitored mass transitions are summarized in Supplementary Table S2. Quality controls consisting of the synthesized apoE isoform-specific peptides (each 15 µmol/L in ammonium bicarbonate buffer) were run in parallel with the samples to verify phenotypes. The isoform-specific peptide standards were provided by the Leiden University Medical Center (Leiden, The Netherlands) within the IFCC apolipoprotein reference candidate method project.

### 2.3. Laboratory Analyzes

Genomic DNA was extracted from peripheral blood leukocytes using an automated protocol on the QIAGEN Autopure LS (QIAGEN, Hilden, Germany). DNA purity and yield were determined on a NanoDrop spectrophotometer. APOE genotyping was performed by a melting curve analysis on a LightCycler^®^ 480 (Hoffman-La Roche, Basel, Switzerland) according to the method of Aslanidis and Schmitz [27].

Glucose and hemoglobin A1c (HbA1c), triglycerides (TG), total cholesterol, LDL-cholesterol (LDL-CH), and HDL-cholesterol (HDL-CH) were determined by automatic assays (Roche Cobas 6000/8000, Roche Diagnostics, Mannheim, Germany), while VLDL concentration was indirectly estimated (triglyceride concentration/2.2).

### 2.4. Statistical Analysis

Statistical analyses were performed with IBM SPSS Statistics (version 20). Samples with mismatches between apoE phenotype (defined by the developed LC-MS/MS assay) and *APOE* genotype were excluded from data evaluation and statistical analysis. Distributions of continuous parameters were described as medians (interquartile ranges) unless otherwise stated. *p* values < 0.05 were considered statistically significant. Basic group comparison (baseline cohort characteristics) was performed with the Mann–Whitney U test for continuous and χ2 test for dichotomous variables. Apolipoprotein and lipid concentrations were ln-transformed (except for LDL-CH) to approximate normal distribution. Linear multiple regression analysis was used to investigate the influence of age, sex, statin therapy and apoE phenotype (according to the peptide-specific phenotype signature, in an isoform dose-dependent manner, reflecting allele-doses) on apoE concentrations. Similarly, linear multiple regression analysis was performed adjusting for age, sex and statin treatment to investigate the influence of apoE phenotype on apolipoprotein and lipid concentrations. We also tested for an interaction of apoE and statin therapy.

## 3. Results

### 3.1. Method Development and Validation

In order to enable simultaneous apo quantification and apoE phenotyping, our on-line SPE-LC-MS/MS setup for quantitative apolipoprotein profiling was optimized in three aspects: (1) alkylation reagent IAA was replaced by NEM; (2) the injection volume was increased from 3 to 5 µL; (3) the mass spectrometric detection was extended by mass transitions of the apoE isoform-specific peptides (Supplementary Table S2). The total run time was 6.5 min. Supplementary Figure S1 shows examples of apoE phenotyping by LC-MS/MS, which was performed by qualitative analysis of four isoform-specific peptides: CLAVYQAGAR (apoE2), LGADMEDVCGR (apoE2 and apoE3), LAVYQAGAR (apoE3 and apoE4) and LGADMEDVR (apoE4) (see Supplementary Table S2). The apoE phenotype could be determined by its characteristic isoform-specific peptide pattern.

Baseline cohort characteristics and results of apolipoprotein quantification are presented in Supplementary Table S1. Phenotyping by LC-MS/MS and genotyping by real-time fluorescence PCR matched in 493 (97.8%) of the initial cases in the CAD cohort and in 214 (97.3%) in the MCI cohort.

### 3.2. ApoE Concentration and apoE Phenotyping in Stable CAD

**Figure 1:**
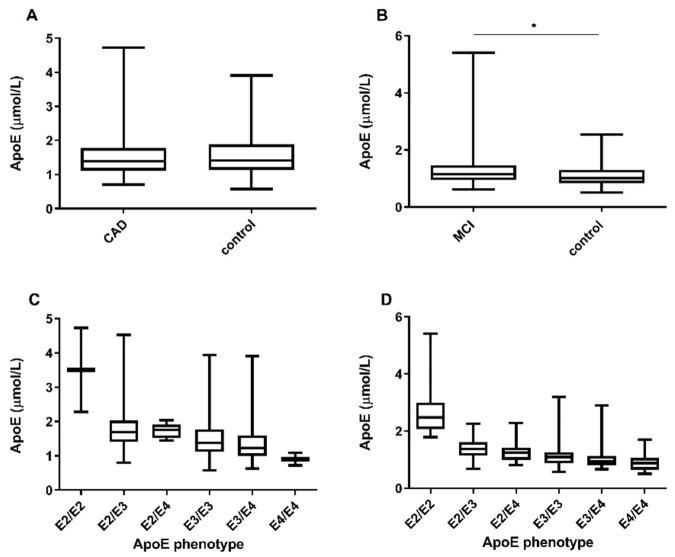
Total apolipoprotein E concentration in relation to cases/control status of our two cohorts (**A**) LIFE-Heart (CAD cohort) and (**B**) LIFE-Adult (MCI cohort). In the CAD study, total apoE levels did not differ between cases and controls (**A**), while in the MCI study total apoE significantly differed between MCI subjects and control group (**B**). ApoE concentrations determined in the six apoE phenotypes are depicted as box-and-whisker plots (min to max value) for the CAD (**C**) and MCI (**D**) cohort. * *p* < 0.05 was considered significant.

Levels of apoE did not differ significantly between CAD patients and the control group, as shown in Figure 1A. In the CAD cohort, age and sex did not significantly affect apoE concentration, while statin therapy significantly lowered apoE concentration by 12% (*p* < 0.001). The apoE phenotype distribution is summarized in Supplementary Table S3. E3/E3 was the most frequent phenotype (61.5%), followed by E3/E4 (21.5%), E2/E3 (14.6%), E2/E4 (1.9%), E2/E2 (0.4%) and E4/E4 (0.4%). In total, 16.8% of the individuals were apoE2 carriers and 23.5% were apoE4 carriers. Individual phenotype frequency did not significantly differ between cases and controls. ApoE2 phenotype was associated with significantly higher total apoE concentration compared to apoE3 and apoE4 (*p* < 0.001) phenotypes, while the difference between apoE3 and apoE4 was not significant (Supplementary Table S4).

### 3.3. ApoE Phenotype Dependent Distribution of Apolipoprotein and Lipid Levels in Stable ASCD

**Figure 2:**
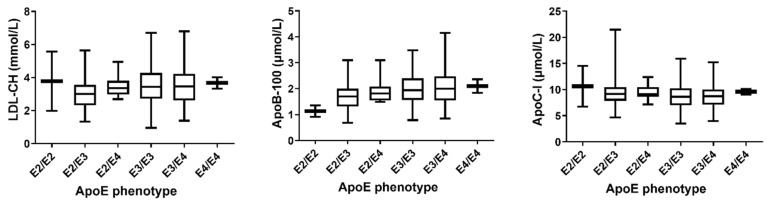
ApoE phenotype-dependent distribution of LDL-cholesterol, apolipoprotein B-100 and apolipoprotein C-I in stable coronary artery disease (CAD) cohort, presented as box-and-whisker plots.

ApoB-100 and apoC-I showed differential distribution between apoE isoforms (Figure 2). Levels of apoB-100 differed between all three isoforms; apoE2 was associated with lower and apoE4 with higher levels compared to apoE3 (apoE2 vs. apoE3 *p* < 0.001, apoE2 vs. apoE4 *p* < 0.001, and apoE3 vs. apoE4 *p* = 0.007; Supplementary Table S5). By contrast, apoE2 was associated with higher apoC-I levels compared to apoE3 (*p* < 0.05), while differences between other isoforms were not significant regarding apoC-I concentration. In addition, apoE2 was associated with lower LDL-cholesterol in comparison to apoE3 (*p* < 0.001, B = −0.513) and apoE4 (*p* < 0.001, B = −0.713), see Supplementary Table S5.

ApoE phenotype-dependent concentrations of total apoE, apoB-100, apoC-I and LDL-cholesterol were investigated in a subgroup of CAD patients on statin therapy (*N* = 100), as well as in CAD patients with diabetes (*N* = 86). In the statin subgroup, patients with apoE2 had by 18% lower apoB-100 levels compared to patients with apoE3 and by 26% lower apoB-100 concentrations than patients with apoE4 phenotype (*p* = 0.050 and *p* < 0.01, respectively). On the contrary, apoE levels were significantly higher in individuals with apoE2 than in individuals with apoE3 (+23%) and apoE4 (+34%) isoforms (*p* < 0.05 and *p* < 0.01, respectively). Levels of apoC-I and LDL-cholesterol did not significantly differ between apoE phenotypes (*p* > 0.05).

Supplementary Table S6 summarizes the significant changes between apoE phenotypes in the statin and diabetic subgroups. Among the diabetic CAD patients, apoC-I levels were 16% higher in the apoE2 phenotype compared to the apoE3 phenotype (*p* < 0.05). In addition, apoE had higher levels in apoE2 than in apoE3 (+27%) and apoE4 (+42%) phenotypes, as observed in the patients with statin therapy (*p* < 0.01, B = 1.270 and *p* < 0.01, B = 1.423). ApoB-100 was not significantly different between the three phenotypes (*p* > 0.05). Likewise, LDL-cholesterol was not significantly different between apoE isoforms in the diabetic subgroup.

### 3.4. ApoE Phenotyping in the MCI Sub-Cohort

In subjects with MCI apoE, concentrations were significantly higher compared to controls (*p* = 0.024) (Figure 1B). In total, 43.5% apoE2 and 50.0% apoE4 carriers were found (Supplementary Table S3). ApoE levels in the MCI cohort were significantly different between all three isoforms: with higher concentrations associated to apoE2 than apoE3 (*p* < 0.001, B = 1.366) and apoE4 (*p* < 0.001, B = 1.536), as well as to apoE3 vs. apoE4 (*p* < 0.001, B = 1.125). There was a pattern of tendency towards a phenotype-dependent decrease in apoE concentration as follows: E2/E2 > E2/E3 > E2/E4 > E3/E3 > E3/E4 > E4/E4 (Figure 1D). Similar observations as in the stable CAD were found in the MCI cohort for the association between apoE isoforms and apoB-100 and cholesterol levels (Supplementary Table S5). Additionally, apoE3 was associated with lower LDL-cholesterol than apoE4 (apoE3 vs. apoE4, *p* = 0.034). In this cohort, HDL-cholesterol concentration was significantly higher in apoE2 compared to apoE4 (*p* = 0.026) (see Supplementary Figure S2). There was a minor age-related impact on total apoE concentration (*p* = 0.027, B = 0.992), while sex and statin therapy did not affect apoE levels significantly (Supplementary Table S4).

**Figure 3:**
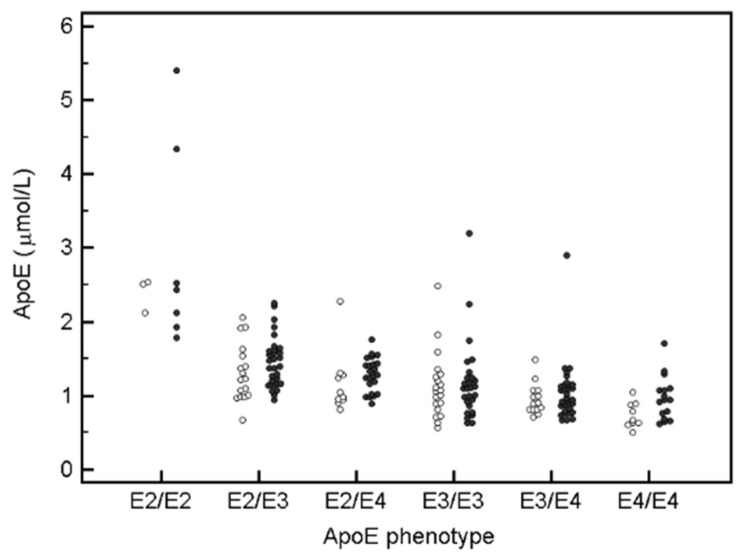
ApoE concentrations for control cases (white dots) and MCI subjects (dark grey dots) within each phenotype.

Significant difference of apoE concentration observed between MCI subjects and controls were further investigated within each phenotype. As shown in Figure 3, there were tendencies towards higher apoE levels in MCI cases compared to controls for each phenotype. Mean apoE concentration (µmol/L) ± SD within each phenotype is as follows: E2/E2 controls (*n* = 3) 2.39 ± 0.237, MCI cases (*n* = 7) 2.94 ± 1.384; E2/E3 controls (*n* = 17) 1.32 ± 0.389, MCI (*n* = 31) 1.45 ± 0.342; E2/E4 controls (*n* = 10) 1.18 ± 0.422, MCI (*n* = 23) 1.28 ± 0.229; E3/E3 controls (*n* = 20) 1.14 ± 0.448, MCI (*n* = 27) 1.18 ± 0.534; E3/E4 controls (*n* = 14) 0.956 ± 0.210, MCI (*n* = 32) 1.05 ± 0.395; E4/E4 controls (*n* = 9) 0.74 ± 0.173, MCI (*n* = 16) 0.97 ± 0.295.

## 4. Discussion

In the present study, we included apoE phenotyping in an established high-throughput mass spectrometric apolipoprotein profiling setup and validated this approach in patients with stable CAD. Using this novel approach, we investigated the impact of apoE phenotypes on apolipoprotein and lipid profiles in MCI study subjects.

While several publications on LC-MS/MS methods for identification or quantification of apoE phenotypes were already published, only a few studies combine apoE phenotyping and quantification of multiple plasma apolipoproteins within one method [17,20]. Our application enables a simultaneous determination of diagnostically relevant parameters from a very small sample volume (3 µL of serum/plasma) and provides both, apolipoprotein level and apoE risk assessment within a single LC-MS/MS run of only 6.5 min. The hands-on time required for the preparation is limited to less than 2 h, emphasizing the potential for a diagnostic routine application. To obtain the same information, it would be required to perform PCR-based genotyping plus eight single apolipoprotein immunoassays. The advantages of such a multiplexed method as a candidate reference method for apolipoprotein quantitation and phenotyping using LC-MS/MS have also been recognized [28]. In our study, 98% agreement of apoE phenotype identified by LC-MS/MS and *APOE* genotyping was achieved which is comparable to previously published results [15]. Since phenotyping and genotyping were not performed from the same sample aliquot in our study, we assume that sample mislabeling, either at sample collection or sample aliquoting, is the main reason for discrepancies.

In stable CAD, we could confirm the known apoB-100 association with apoE phenotypes. Out of the other seven quantified apos, the apoE phenotype affected only apoC-I. The interaction between apoE and apoC-I is already described. Mediated by apoE, apoC-I inhibits the binding of remnants by the LDL receptor-related protein [29,30]. Higher concentrations of apoC-I in VLDL have been observed in patients with ischemic heart disease and those with greater postprandial lipemia [31]. Structure/function studies revealed that apoE2 is severely defective in LDL receptor binding [32]. Here we found 16% higher apoC-I levels in apoE2 compared to apoE3 carriers in our diabetic CAD patients, but not in the statin treated CAD patients. Higher apoC-I levels were previously described in the ε2/ε3 genotype compared to the ε3/ε4 genotype in normolipemic study subjects [33]. However, in the subgroup of CAD patients on statin therapy, apoC-I and LDL-cholesterol were not influenced by the apoE phenotype. Here, apoB-100 concentrations varied between the apoE isoforms, with apoE2 having the lowest levels, significantly different from apoE3 (18% decreased) and apoE4 (26% decreased).

Higher apoE but not apoB-100 levels were observed in patients with MCI in this study, according to results from other studies [34]. We confirmed with our study that mild cognitive impairment is associated with *APOE4* genotype. Moreover, we found differences in apoE plasma concentrations observed between apoE2 and apoE3/apoE4 accounting for about 30% of its variation, with the lowest levels observed in apoE4 and the highest levels in apoE2 phenotypes, as observed previously [13,14,35,36]. This can be explained by lower apoE2 isoform affinity towards the LDL-receptor compared to apoE3 and apoE4 isoforms, leading to higher apoE concentrations [32].

The main limitation of our study is the small study size. While our observations imply apoE phenotype-dependent variations of apoB-100/apoC-I in the respective ASCVD subgroups, it is important to note that the number of statin-treated and diabetic patients was limited to 100 and 86, respectively. Therefore, in future investigations correlations between apoE phenotype and apoB-100/apoC-I levels should be proven in larger study cohorts. This can be now achieved under routine conditions by application of our rapid combined apoliprotein quantification and apoE phenotyping.

## 5. Conclusions

This study demonstrates a successful high-throughput application of simultaneous apolipoprotein profiling and apoE phenotyping from 3 µL of human plasma. We observed the apoE isoform’s influence on plasma lipid and apolipoprotein levels. The ApoE phenotype affected total apoE levels in both investigated cohorts. Specifically, for apoB-100, an apoE phenotype-dependent effect was observed in CAD patients under statin therapy, while in diabetic CAD patients apoC-I concentration was apoE phenotype-dependent. Future studies should examine the role of individual apoE phenotypes in relation to apoB-100 and apoC-I in patients with cardiometabolic syndrome.

## Data Availability

The data presented in this study are available in the article and in the Supplementary Materials section. Any other details are available on request from the corresponding author.

## Supplementary Materials

The following supporting information can be downloaded at: https://www.mdpi.com/article/10.3390/nu14122474/s1, Figure S1:Representative chromatograms of apoE phenotype-specific peptides and determined phenotypes in CAD cohort; Figure S2: ApoE phenotype-dependent total cholesterol (t-cholesterol) and HDL-cholesterol (HDL-CH) levels in coronary artery disease (CAD) and mild cognitive impairment (MCI) cohort; Table S1: Baseline characteristics of the case-control subgroups of Leipzig LIFE Heart Study (*n* = 481) and LIFE Adult Study (*n* = 209); Table S2: Mass spectrometry parameters of the investigated proteotypic peptides; Table S3: ApoE phenotype distribution in coronary artery disease (CAD) and mild cognitive impairment (MCI) cohorts; Table S4: Influence of age, sex, statin therapy and apoE isoforms on total apoE level in coronary artery disease (CAD) and mild cognitive impairment (MCI) cohort; Table S5: Influence of apoE isoforms on apolipoprotein and lipid levels; Table S6: Significant apoE phenotype comparisons for total apoE, apoB100 and apoC-I in coronary artery disease patients under statin therapy or with diagnosed diabetes mellitus.
