# Supplementary material for: Simultaneous Mass Spectrometry-Based Apolipoprotein Profiling and Apolipoprotein E Phenotyping in Patients with ASCVD and Mild Cognitive Impairment
Source: Nutrients. 2022 Jun 15;14(12):2474. doi: 10.3390/nu14122474 (PMC9230692; doi:10.3390/nu14122474)
Supplement: Supplementary file 1 [file nutrients-14-02474-s001.zip › nutrients-1751194-supplementary.pdf]

Supplementary Material

Simultaneous Mass Spectrometry-Based Apolipoprotein Profiling and Apolipoprotein E Phenotyping in Patients with ASCVD and Mild Cognitive Impairment

Ilijana Begcevic Brkovic [1], Benedikt Zöhrer [2], Markus Scholz [3,4], Madlen Reinicke [1], Julia Dittrich [1], Surab Kamalsada [1], Ronny Baber [1,4], Frank Beutner [4,5], Andrej Teren [4,6], Christoph Engel [3,4], Kerstin Wirkner [3,4], Holger Thiele [5], Markus Löffler [3,4], Steffi G. Riedel-Heller [7] and Uta Ceglarek [1,4,*]

Detailed Methods

Reagents and Chemicals

Ammonium bicarbonate, *N*-Ethylmaleimide (NEM), 2,2,2-Trifluoroethanol, *tris*(2-carboxyethyl)-phosphine and tosyl phenylalanyl chloromethyl ketone treated bovine trypsin were purchased from Sigma Aldrich (St. Louis, MO, USA) whereas iodoacetamide (IAA) and formic acid were acquired from Fluka (Buchs, Switzerland). ULC/MS grade methanol, acetonitrile and 2-propanol were purchased from Biosolve (Valkenswaard, the Netherlands). Ultra-pure water from a Barnstead NANOpure water purification system (Thermo Fisher Scientific, Waltham, MA, USA) was used. LC grade ethanol was purchased from Merck (Darmstadt, Germany). Sequencing grade modified porcine trypsin was acquired from Promega (Madison, WI, USA).

Synthetic peptides

Peptides were synthesized by the Core Unit Peptide Technologies Leipzig (Faculty of Medicine, Leipzig University). Lyophilized standards of stable isotope labeled (SIL) and unlabeled synthetic peptides were stored at -20 °C. Peptide stock solutions were prepared in 2-propanol/water (1:1, v/v) and diluted with 100 mmol/L ammonium bicarbonate to obtain working standard solutions which were stored at - 80 °C. ApoE isoform specific peptide standards were provided by the Leiden University Medical Center (The Netherlands).

Sample Preparation

An internal standard (IS) mix containing SIL peptides in 100 mmol/L ammonium bicarbonate was added to 3 µL aliquots of calibration standards, quality controls or plasma resulting in a two-fold dilution. Protein denaturation was performed using 6.9 mol/L 2,2,2-Trifluoroethanol. Samples were reduced within 30 min by incubation with 5 mmol/L *tris*(2-carboxyethyl)phosphine at 60 °C. Alkylation was performed in 30 min at room temperature using either 10 mmol/L *N*-Ethylmaleimide or 10 mmol/L iodoacetamide. After a ten-fold dilution with 100 mmol/L ammonium bicarbonate and subsequent

addition of 8 µg trypsin dissolved in 50 mmol/L acetic acid, the tryptic digestion performed at 37 °C was completed in 16 h. After acidification with 0.1% formic acid, the samples were centrifuged for 5 min at 13,000 rpm. The supernatant was transferred to an autosampler vial and stored at -80 °C until analysis.

LC-MS/MS analysis

The temperature of the autosampler and the oven were 10 °C and 40 °C, respectively. 3 µL were injected into the LC-MS/MS system and sample purification was achieved by on-line SPE on a Strata-X On-Line Extraction column (20 x 2 mm i.d., 25 µm particle size) from Phenomenex (Torrance, CA, USA). Sample loading and washing were performed in 0.21 min using 0.02% formic acid in water/methanol (90/10, v/v) at 1.5 mL/min. After automated column switching and back-flush elution onto a ZORBAX 300SB-C18 column (150 x 1.0 mm i.d., 3.5 µm particle size) with corresponding guard column (Agilent Technologies, Santa Clara, CA, USA) the chromatographic separation was completed within a total run time of 6.5 min. The mobile phase consisted of 0.1% formic acid in water/methanol (90/10, v/v) as eluent A and 0.1% formic acid in water/methanol (10/90, v/v) as eluent B. Gradient elution at a flow rate of 50 µL/min was performed as follows: 0-0.21 min at 10% B, 0.21-1.48 min linear increase to 60% B, 1.48-2.48 min linear increase to 70% B, 2.48-2.68 min linear increase to 100% B, 2.68-6.10 min at 100% B, re-equilibration from 6.20-6.50 min with 10% B. Simultaneously to the chromatographic separation on the analytical column, the SPE column was rinsed with acetonitrile/isopropanol (1:1, v/v) at 1.5 mL/min from 6.10-6.40 min prior to re-equilibration with 0.02% formic acid in water/methanol (90/10, v/v).

To avoid unnecessary contaminations of the mass spectrometer, a diverter valve was used to discard the eluent stream outside the elution window into waste. MRM transitions are summarized in Table S2. The Turbo V™ ion spray source was operated with the following settings: ion spray voltage = 5500 V, ion source heater temperature = 450 °C, collision gas = 9, source gas 1 = 40 psi, source gas 2 = 50 psi, curtain gas = 40 psi, unit resolution of Q1 and Q3.

Chromatographic conditions and mass spectrometric detection were controlled by Chromeleon™ 6.80 software (Thermo Scientific Dionex) and Analyst® 1.5.1 (SCIEX, Framingham, MA, USA), respectively.

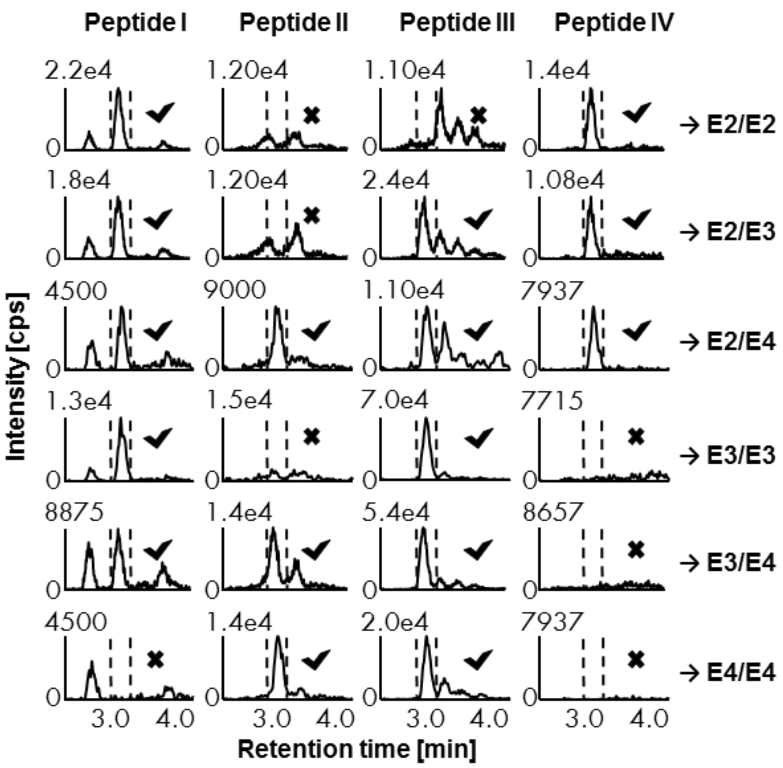

Supplementary Figure S1. Representative chromatograms of apoE phenotype-specific peptides and determined phenotypes in CAD cohort. Based on qualitative examination of four peptides (peptide I: LGADMEDVCGR, peptide II: LGADMEDVR, peptide III: LAVYQAGAR, peptide IV: CLAVYQAGAR) and its pattern of presence, apoE phenotypes were identified.

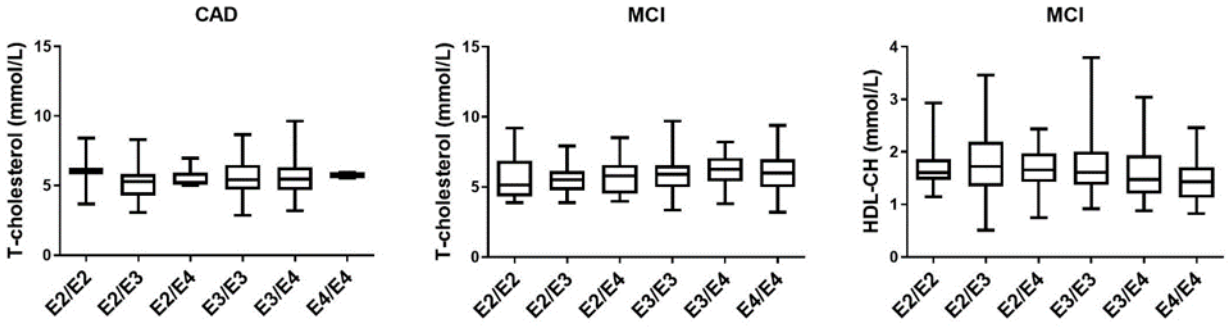

Supplementary Figure S2. ApoE phenotype-dependent total cholesterol (t-cholesterol) and HDL-cholesterol (HDL-CH) levels in coronary artery disease (CAD) and mild cognitive impairment (MCI) cohort, presented as box and whiskers plots. Significant differences were observed for t-cholesterol in CAD cohort between: apoE2 and apoE3; apoE2 and apoE4 phenotype ($p=0.002$, $B=0.927$ and $p<0.001$, $B=0.890$, respectively), as well as in the MCI cohort apoE2 vs. apoE3; apoE2 vs. apoE4 ($p=0.009$, $B=0.939$ and $p=0.002$, $B=0.921$, respectively) phenotype. HDL-CH concentrations differed between apoE2 and apoE4 in MCI cohort ($p=0.026$, $B=1.088$).

Supplementary Table S1. Baseline characteristics of the case-control subgroups of Leipzig LIFE Heart Study (n=481) and LIFE Adult Study (n=209).

	**Stable coronary artery disease**				**Mild cognitive impairment (SISCO)**			
	Overall	Controls	**Cases**	***p* value**	**Overall**	**Controls**	**Cases**	***p* value**
Number of cases	481	221	260		209	73	136	
Men, %	59	45	70	< 0.001	47	51	45	0.448
Age, yrs	65 (54-72)	63 (53-71)	66 (56-73)	0.024	69 (65-73)	69 (65-73)	69 (65-73)	0.924
SISCO						53 (53-54)	45 (43-49)	< 0.001
MMSE						30 (29-30)	27 (26-28)	< 0.001
Body mass index, kg/m2	28.8 (26.4-32.8)	28.8 (26.5-32.8)	29.0 (26.3-32.5)	0.951	27.4 (25.5-30.1)	26.7 (24.7-28.9)	27.8 (25.6-30.5)	0.094
Diabetes mellitus, %	30	25	33	0.049	31	21	37	0.048
Current smoker, %	16	11	20	0.007	12	13	11	0.777
Statin therapy, %	34	30	39	0.048	17	17	17	0.918
Fasting, %	29	25	32	0.130	100	100	100	1.000
Single-vessel disease (n [%])	133 (51.2)		133 (51)					
Double-vessel disease (n [%])	89 (34.2)		89 (34)					
Triple-vessel disease (n [%])	38 (14.6)		38 (15)					
Total cholesterol, mmol/L	5.40 (4.65-6.36)	5.32 (4.77-6.00)	5.44 (4.55-6.54)	0.233	5.82 (4.86-6.61)	5.83 (4.94-6.69)	5.75 (4.77-6.58)	0.504
HDL cholesterol, mmol/L	1.28 (1.07-1.54)	1.38 (1.13-1.61)	1.20 (1.02-1.47)	< 0.001	1.59 (1.27-1.93)	1.69 (1.41-2.01)	1.55 (1.22-1.86)	0.021
LDL cholesterol, mmol/L	3.33 (2.65-4.15)	3.24 (2.60-3.92)	3.48 (2.71-4.40)	0.018	3.69 (2.78-4.45)	3.70 (2.90-4.56)	3.69 (2.67-4.42)	0.559
VLDL cholesterol, mmol/L	0.76 (0.55-1.14)	0.74 (0.52-1.13)	0.80 (0.57-1.15)	0.198				
Triglycerides, mmol/L	1.70 (1.22-2.51)	1.62 (1.15-2.50)	1.78 (1.25-2.56)	0.228	1.31 (0.89-1.69)	1.22 (0.88-1.46)	1.41 (0.90-2.13)	0.033

Creatinine, µmol/L	77 (65-88)	74 (63-86)	79 (68-89)	0.005	79 (69-90)	79 (70-90)	79 (67-91)	0.856
Glucose, mmol/L	6.04 (5.30-7.35)	5.76 (5.23-6.84)	6.26 (5.41-7.61)	0.001	5.65 (5.29-6.28)	5.57 (5.19-6.00)	5.79 (5.35-6.40)	0.005
HbA1c, %	5.7 (5.4-6.1)	5.6 (5.4-6.0)	5.8 (5.4-6.3)	0.022	5.5 (5.2-5.8)	5.4 (5.2-5.7)	5.5 (5.3-5.9)	0.014
apo A-I, µmol/L	57.7 (50.1-66.0)	59.5 (52.1-67.2)	56.3 (48.6-64.0)	0.001	55.3 (47.6-64.5)	56.9 (49.4-66.1)	53.9 (46.7-63.9)	0.22
apo A-II, µmol/L	47.1 (41.3-53.9)	47.6 (41.8-54.6)	46.8 (40.8-53.3)	0.218	48.5 (41.8-56.4)	47.5 (41.2-57.0)	48.9 (42.3-56.2)	0.857
apo A-IV, µmol/L	2.50 (1.94-3.16)	2.55 (2.02-3.20)	2.47 (1.88-3.12)	0.244	2.11 (1.75-2.66)	2.11 (1.77-2.68)	2.12 (1.74-2.64)	0.931
apo B-100, µmol/L	1.88 (1.53-2.35)	1.80 (1.44-2.24)	1.97 (1.63-2.41)	0.002	1.49 (1.22-1.90)	1.45 (1.21-1.95)	1.49 (1.22-1.89)	0.803
apo C-I, µmol/L	8.73 (7.15-10.2)	8.84 (7.50-10.3)	8.60 (6.91-10.1)	0.080	8.18 (6.93-9.39)	8.05 (6.20-9.25)	8.22 (7.15-9.50)	0.413
apo C-III, µmol/L	18.6 (16.1-22.5)	18.7 (16.1-23.1)	18.6 (16.1-22.2)	0.462	14.0 (10.8-17.4)	12.8 (10.0-16.9)	14.6 (11.0-17.7)	0.188
apo E, µmol/L	1.4 (1.1-1.8)	1.4 (1.1-1.9)	1.4 (1.1-1.8)	0.307	1.12 (0.91-1.40)	1.03 (0.82-1.31)	1.16 (0.94-1.46)	0.024
apo J, µmol/L	4.81 (4.23-5.55)	4.84 (4.23-5.49)	4.77 (4.23-5.61)	0.989	3.71 (3.21-4.20)	3.59 (3.22-4.17)	3.75 (3.21-4.23)	0.994

Values are presented as median (interquartile range) unless otherwise stated. p values of continuous and dichotomous variables were calculated using Mann-Whitney U test and χ^2 test, respectively. apo, apolipoprotein; CAD, coronary artery disease; HbA1c, hemoglobin A1c; HDL, high-density lipoprotein; LDL, low-density lipoprotein; VLDL, very low-density lipoprotein.

Supplementary Table S2. Mass spectrometry parameters of the investigated proteotypic peptides.

Peptide Sequence	Protein	apoE Phenotyping Peptide	Precursor *m/z*	DP (V)	Product ion	Product ion *m/z*	CE (V)
ATEHLST**L**SEK	apoA-I		608.2	100	y_7	777.4	34
SPE**L**QAEAK	apoA-II		486.7	105	y_6	659.3	27
S**L**APYAQDTQEK	apoA-IV		675.7	100	y_9^{2+}	540.2	28
FPEVDV**L**TK	apoB-100		524.2	90	y_8^{2+}	450.6	28
TPDVSSA**L**DK	apoC-I		516.6	90	y_6	620.2	29
TY**L**PAVDEK	apoC-II		518.2	75	y_6	658.2	23
GWVTDGFSS**L**K	apoC-III		598.7	80	y_9	953.3	24
NI**L**TSNNIDVK	apoD		615.7	75	y_8	890.3	25
LGP**L**VEQGR	apoE		484.7	80	y_5	588.2	29
LGADMEDVC[CAM]GR	non-apoE4	I	611.8	94	+2y4	491.2	43
					+2y5	606.3	37
LGADMEDVC[NEM]GR	non-apoE4	I	645.8	94	+2y4	559.3	43
					+2y5	674.3	37
LGADMEDVR	E4	II	503.2	78	+2y2	274.2	36
					+2y8	892.4	25
LAVYQAGAR	non-apoE2	III	474.8	66	+2b2	185.1	23
					+2y6	502.3	24
C[CAM]LAVYQAGAR	E2	IV	554.8	71	+2y6	665.3	23
					+2y8	835.4	32
C[NEM]LAVYQAGAR	E2	IV	588.8	71	+2y6	665.3	23
					+2y8	835.4	32
AT**V**VYQGER	apoH		511.7	80	y_6	751.3	25
ASSIIDE**L**FQDR	apoJ		697.2	85	y_7	922.2	34
SLTSC[NEM]**L**DSK	apoM		539.7	90	y_7	878.2	22

Bold and underlined amino acids indicate the position of ($^{13}C_6{}^{15}N$)Leu or ($^{13}C_5{}^{15}N$)Val in the SIL peptides. Identical transitions were measured for SIL peptides in due consideration of the expected mass shift. [CAM] denotes the measurement of a carbamidomethylated cysteine, i.e. +57 molecular mass, whereas [NEM] indicates N-ethylmaleimide (NEM)-modified cysteine, i.e. +125 molecular mass. For apoE phenotyping two transitions per peptide were monitored. Dwell time was set to 10 ms. DP, declustering potential; CE, collision energy.

Supplemental Table S3. ApoE phenotype distribution in coronary artery disease (CAD) and mild cognitive impairment (MCI) cohorts.

ApoE phenotype	CAD cohort (n=481)	MCI cohort (n=209)
E2/E2, n (%)	2 (0.4)	10 (4.8)
E2/E3, n (%)	70 (14.6)	48 (23.0)
E2/E4, n (%)	9 (1.9)	33 (15.8)
E3/E3, n (%)	296 (61.5)	47 (22.5)
E3/E4, n (%)	102 (21.2)	46 (22.0)
E4/E4, n (%)	2 (0.4)	25 (12.0)
E2 carriers, n (%)	81 (16.8)	91 (43.5)
E4 carriers, n (%)	113 (23.5)	104 (50.0)

Supplementary Table S4. Influence of age, sex, statin therapy and apoE isoforms on total apoE level in coronary artery disease (CAD) and mild cognitive impairment (MCI) cohort.

	CAD cohort		MCI cohort	
**Variable**	**B**	***p*-value**	**B**	***p*-value**
**Age**	0.999	0.283	0.992	0.027
**Sex**	0.993	0.823	1.067	0.108
**Statin therapy**	0.883	<0.001	0.916	0.107
**ApoE isoforms:**				
apoE2 vs. apoE3	1.245	<0.001	1.366	<0.001
apoE2 vs. apoE4	1.323	<0.001	1.536	<0.001
apoE3 vs. apoE4	1.063	0.086	1.124	<0.001

B, unstandardized regression coefficient (given as antilogarithm).

Supplementary Table S5. Influence of apoE isoforms on apolipoprotein and lipid levels.

	CAD cohort		MCI cohort	
**Isoform**	**B coefficient**	***p*-value**	**B coefficient**	***p*-value**
**apoA-I**				
ApoE2 vs. ApoE3	1.001	0.997	1.037	0.185
ApoE2 vs. ApoE4	1.005	0.874	1.044	0.145
ApoE3 vs. ApoE4	1.004	0.849	1.006	0.787
**apoA-II**				
ApoE2 vs. ApoE3	0.998	0. 923	0.980	0.509
ApoE2 vs. ApoE4	1.008	0. 785	0.979	0.530
ApoE3 vs. ApoE4	1.011	0.622	1.000	0.992
**apoA-IV**				
ApoE2 vs. ApoE3	1.059	0.190	1.021	0.570
ApoE2 vs. ApoE4	1.013	0.808	1.017	0.680
ApoE3 vs. ApoE4	0.957	0.261	0.995	0.879
**apoB-100**				
ApoE2 vs. ApoE3	0.834	<0.001	0.817	<0.001
ApoE2 vs. ApoE4	0.770	<0.001	0.764	<0.001
ApoE3 vs. ApoE4	0.923	0.007	0.936	0.047
**apoC-I**				
ApoE2 vs. ApoE3	1.068	0.032	1.047	0.189
ApoE2 vs. ApoE4	1.048	0.228	1.048	0.207
ApoE3 vs. ApoE4	0.981	0.479	1.001	0.970
**apoC-III**				
ApoE2 vs. ApoE3	1.002	0.944	0.972	0.563
ApoE2 vs. ApoE4	0.984	0. 695	0.956	0.391
ApoE3 vs. ApoE4	0.982	0. 528	0.984	0.690
**apoJ**				
ApoE2 vs. ApoE3	0.966	0.145	1.011	0.652
ApoE2 vs. ApoE4	0.953	0.109	0.978	0.396
ApoE3 vs. ApoE4	0.987	0.530	0.967	0.109
**HDL-CH**				
ApoE2 vs. ApoE3	1.033	0. 291	1.038	0.298
ApoE2 vs. ApoE4	1.074	0.072	1.088	0.026
ApoE3 vs. ApoE4	1.039	0.175	1.048	0.113
**LDL-CH**				
ApoE2 vs. ApoE3	-0.513	<0.001	-0.425	0.001
ApoE2 vs. ApoE4	-0.713	<0.001	-0.653	<0.001
ApoE3 vs. ApoE4	-0.200	0.053	-0.227	0.034
**VLDL-CH**				
ApoE2 vs. ApoE3	1.030	0. 626	na	na
ApoE2 vs. ApoE4	0.971	0.705	na	na
ApoE3 vs. ApoE4	0.942	0.272	na	na
**Total-CH**				
ApoE2 vs. ApoE3	0.927	0.002	0.939	0.009
ApoE2 vs. ApoE4	0.890	<0.001	0.921	0.002
ApoE3 vs. ApoE4	0.961	0.067	0.981	0.361

TG				
ApoE2 vs. ApoE3	1.039	0.530	1.021	0.777
ApoE2 vs. ApoE4	0.982	0.816	0.940	0.441
ApoE3 vs. ApoE4	0.945	0.303	0.920	0.201

B, unstandardized regression coefficient (given as antilogarithm, except for LDL-CH); na, not available; HDL-CH, high-density lipoprotein cholesterol; LDL-CH, low-density lipoprotein cholesterol; VLDL-CH, very low-density lipoprotein cholesterol; total-CH, total cholesterol; TG, triglycerides.

Supplementary Table S6. Significant apoE phenotype comparisons for total apoE, apoB100 and apoC-I in coronary artery disease patients under statin therapy or with diagnosed diabetes mellitus.

	Statin subgroup (N=100)				Diabetic subgroup (N=86)			
	apoE		apoB-100		apoE		apoC-I	
**Phenotype comparison**	**B coefficient**	**p-value**	**B coefficient**	**p-value**	**B coefficient**	**p-value**	**B coefficient**	**p-value**
ApoE2 vs. ApoE3	1.230	<0.05	0.823	0.05	1.270	<0.01	1.160	<0.05
ApoE2 vs. ApoE4	1.344	<0.01	0.739	<0.01	1.423	<0.01	ns	ns
ApoE3 vs. ApoE4	ns	ns	ns	ns	ns	ns	ns	ns

ns, not significant.
